# Dermal Mitoses Correlate with Surgical Burden in Lentigo Maligna Melanoma: PRAME for Margin Assessment

**DOI:** 10.3390/cancers17193112

**Published:** 2025-09-24

**Authors:** Thomas Leibing, Clara Ziemann, Cyrill Géraud, Jochen Utikal, Sebastian A. Wohlfeil

**Affiliations:** 1 Department of Dermatology, Venereology and Allergology, University Medical Center and Medical Faculty Mannheim, Heidelberg University, 68135 Mannheim, Germany; 2 MVZ Dermatopathologie Prof. Dr. Kind GmbH, 63067 Offenbach, Germany; 3 Section of Clinical and Molecular Dermatology, University Medical Center and Medical Faculty Mannheim, Heidelberg University, 68135 Mannheim, Germany; 4 European Center for Angioscience (ECAS), University Medical Center and Medical Faculty Mannheim, Heidelberg University, 68135 Mannheim, Germany; 5 DKFZ Hector Cancer Institute at the University Medical Center Mannheim, 69120 Heidelberg, Germany; 6 Skin Cancer Unit, German Cancer Research Center (DKFZ), 69120 Heidelberg, Germany

**Keywords:** Lentigo maligna melanoma, PRAME

## Abstract

**Background/Introduction:** Margin assessment in Lentigo maligna (LM) and Lentigo maligna melanoma (LMM) is challenging. Many of these lesions require extensive surgical procedures for R0 resections with unclear histological and clinical features predicting lateral spread. Recently, PRAME was described as a useful antibody to determine margins in these entities. However, several questions, like acceptable PRAME^+^ cell number and density in resection margins and optimal safety margins, especially in head and neck areas, remain. **Methods:** We analyzed cases of LMIS and LMM since the introduction of PRAME and before the introduction of PRAME, with more than 171 cases in total. We re-stained security margins with PRAME. **Results:** We identified a correlation between reported dermal mitoses in LMM and the number of surgical procedures. In many LMM cases before the widespread use of PRAME from our archives, we detected a high density of PRAME^+^ cells in melanoma security margins previously labeled tumor-free. No local recurrence could be identified in these cases, with the caveat of limited follow-up and small case numbers. **Conclusions:** Our findings raise important questions regarding margin assessment with PRAME and the reporting of residual cells in margins.

## Simple Summary

Achieving tumor-free margins in Lentigo maligna and Lentigo maligna melanoma is a significant surgical challenge. In our analysis of over 171 cases, we identified a direct correlation between the presence of dermal mitoses and an increased number of surgical procedures required for clearance. Beyond this, our primary goal was to clarify the clinical significance of PRAME-positive melanocytes at the resection edge. We retrospectively stained archival margins previously classified as negative and found PRAME-positive isolated and grouped cells in some cases. Critically, no local recurrences were observed in these patients (albeit with small numbers and limited follow-up). This finding questions the assumption that any PRAME positivity at a margin necessitates re-excision, highlighting an urgent need to establish a clinically relevant threshold to guide surgical decisions and prevent potential overtreatment.

## 1. Introduction

Malignant melanoma is a common and aggressive skin cancer, with an estimated 325,000 cases worldwide in 2020 [1]. Increasing UV-exposure, causing acute and chronic sun damage (CSD), is likely leading to a steady increase in melanoma incidence, especially in skin types I and II [1]. Since 2018, malignant melanomas have been classified into different pathways by the WHO, with a central focus on CSD [2]. Lentigo maligna melanoma (LMM) is a malignant melanocytic tumor with an invasive dermal component that arises on areas of the body with high CSD. It is mostly found on the head, but also on the arms, and rarely on the trunk of elderly patients [3]. Similarly, its in situ counterpart, Lentigo maligna (LM), also occurs in high CSD regions but lacks dermal invasion of melanocytes histologically. LM is often considered a precursor lesion to LMM [4] while there is ongoing debate on how LM and LMM arise, since there are no defined precursor lesions [4]. Molecularly, LMM does not show *BRAF*^V600E^ or *NRAS* mutations as often as other melanoma subtypes [5,6], which limits therapeutic options in advanced disease stages, although targeted therapy of the common *BRAF*^V600K^ mutation using BRAF/MEK inhibitors can be considered [7]. Due to the high degree of CSD, tumor mutational burden is generally high, which is the only tumor-agnostic United States Food and Drug Administration-approved biomarker for immune checkpoint inhibition [8].

Most guidelines recommend complete resection of primary LM with a safety margin of up to 5 mm. Some authors argue that a 5 mm safety margin might be insufficient, as LM is often clinically hard to spot and its growth pattern is often discontinuous [9,10]. Resection margins of LMM are defined after primary resection, depending on tumor infiltration depth, but at least 10 mm [11,12,13,14,15]. Evidence for these numbers is limited [16,17,18]. Surgically, it is often challenging to achieve tumor-free margins and a cosmetically acceptable result for patients since it often affects sensitive areas like the face. To this end, many surgical techniques (square method, perimeter technique, ‘slow Mohs’, staged radial sections, staged “mapped” excisions, or Mohs micrographic surgery) have been reviewed, with a low number of high-quality studies included [19]. Staged excision and use of immunohistochemistry for all margins has been proposed as a way of minimizing unnecessary surgical morbidity while retaining acceptable margins and keeping local recurrences low [20], since it is histologically often difficult to morphologically distinguish tumor cells from normal melanocytes in the resection borders. Furthermore, it represents a challenge to determine normal melanocyte density in the epidermis on sun-damaged skin, especially in small biopsies without clinico-pathological correlation or re-excisions without the primary excision available for review [21].

PRAME [22] has recently been proposed as a useful marker in distinguishing malignant melanoma from benign melanocytic nevi, especially in the context of acral melanoma and LMIS, as well as LMM [23]. Its usefulness is particularly recognized to distinguish neoplastic melanocytes from PRAME-positive primary tumors from reactive melanocytes in resection borders, especially in LM and LMM [24,25,26,27,28].

In our experience, we often noticed PRAME-positive single cells in resection borders of resected LMs and LMMs in primary excisions and re-excisions of previously resected melanomas, which were not readily detectable in ordinary H&E-stained slides. To assess the role and potential pitfalls of PRAME, we performed a retrospective analysis of those cases.

## 2. Materials and Methods

### 2.1. Case Cohorts

#### 2.1.1. First Cohort

Data of 80 cases with resected LM or LMM from a single tertiary center (Dermatology, Venerology and Allergology, University Medical Centre Mannheim, Heidelberg University) were collected from February 2021 (since the introduction of PRAME in pathological evaluation) to May 2024 (data cut). All cases were routinely stained for PRAME (see below). Data collected included patient age at initial diagnosis, histological subtypes, ulceration, dermal mitosis, localization, history of primary melanoma (Y/N), initial tumor stage, and number of operative procedures to achieve free margins. LM and LMM cases that showed residual tumor in resection borders in more than one repeated resection after the first excision (excluding punch-biopsies for histological confirmation) were defined as Cohort 1.1 (“hard to resect”, 13 cases). LM and LMM cases, which were margin-free in the primary excision or first re-excision after primary excision, were defined as Cohort 1.2 (“easy to resect”, 67 cases). Cohorts were compared regarding the collected data.

#### 2.1.2. Second Cohort

Data of all resected LMM (including LMM cases from the first cohort with available mitoses in pathology reports) from the same institution were collected. A total of 171 cases were identified. The following information was obtained: Breslow thickness, ulceration, dermal mitoses (quantitative), localization, patient age, and number of operative procedures to achieve margin-free resections (R0). Two additional subgroups were formed: Cohort 2.1 (“Dermal mitoses reported”, 31 cases) and Cohort 2.2 (“No dermal mitoses reported”, 140 cases).

### 2.2. Experimental Cohort

Safety margins were taken after micrographically R0 resection of seven cases from Cohort 2.1 and 15 cases from Cohort 2.2, which were previously not stained for PRAME, were re-stained for H&E, PRAME, and SOX10, and re-evaluated for tumor residues in a blinded manner. Junctional nests and single PRAME-positive single and grouped cells were evaluated as “present” or “absent” in both groups. Inclusion criteria for this group were patient consent for additional studies on archived tissue and tissue availability. Melanoma classification was undertaken using the 2018 AJCC guidelines, regardless of date of diagnosis [29].

### 2.3. Protocol for Treatment of LMIS and LMM

Treatment of Lentigo maligna and Lentigo maligna melanoma was performed according to a standard operating procedure adapted after German consensus guidelines regarding melanoma care [30]. All specimens were fixed in 10% formaldehyde and routinely processed for paraffin embedding (FFPE).

### 2.4. Immunohistochemistry

FFPE sections (3 µm) were processed on the Dako Omnis platform (Agilent Technologies, Santa Clara, CA, USA) using the “low” antigen retrieval protocol according to the manufacturer’s instructions using monoclonal antibodies against SOX10 (QR006, Quartett, Potsdam, Germany; 1:100) and PRAME (QR005, Quartett, Potsdam, Germany; 1:100) using a red chromogen and primary PRAME-positive LM/LMM cases as controls in each run.

### 2.5. Imaging and Image Analyses

Imaging was performed using a NanoZoomer S360 Digital slide scanner (Hamamatsu Photonics K.K., Hamamatsu City, Japan). Whole slide images were reviewed and downloaded using PathoZoom Digital Lab (Smart In Media AG, Cologne, Germany). Fiji [31] was used for post-processing (Cropping, Scale bar).

### 2.6. Statistics

All calculations were performed using JMP 16.0 (SAS Institute Inc., Cary, NC, USA). For pairwise comparisons assuming normality, the Student’s *t*-test was used. For pairwise nonparametric comparisons, the Mann–Whitney U test was used. To compare the distribution of categorical data, Pearson’s chi-squared test was used.

## 3. Results

To investigate the correlation of clinical and histologic characteristics of LMM and LM on surgical outcome, we analyzed 80 cases with LM or LMM from February 2021 to May 2024 to screen for relevant characteristics. This group was further separated into two cohorts: “hard to excise” with more than one re-excision, and “easy to excise” with maximum of one repeated excision after primary tumor resection (Table 1).

**Figure 1:**
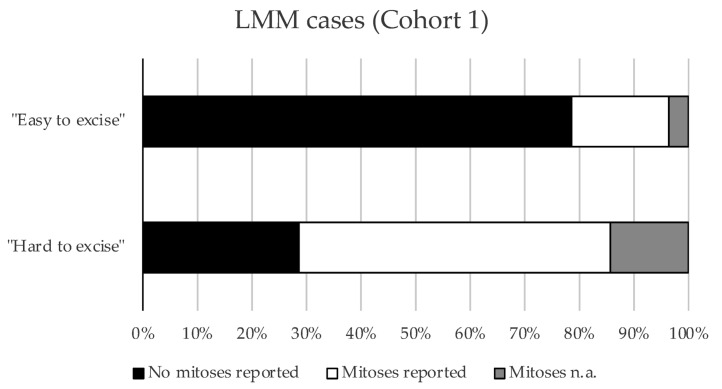
Mitoses in respective subgroups of Cohort 1. Bar graph showing the proportions of cases with or without mitoses in the “easy to excise” and “hard to excise” LMM cases from Cohort 1 (*p* = 0.0382, Pearson’s chi-squared test).

There were no significant differences between the “hard to resect” and “easy to resect” subgroups, testing all parameters collected except for “Excisions for R0”. Upon subgrouping for stage, we found that “hard to resect” LMM cases were significantly more often associated with detectable (≥1) dermal mitosis in LMM compared to the “easy to resect” LMM cases (*p* = 0.0382) (Figure 1).

To validate these findings, we extended our cohort and analyzed 171 cases with LMM that were treated between 1 June 2011 and 31 May 2024 (Table 2).

**Figure 2:**
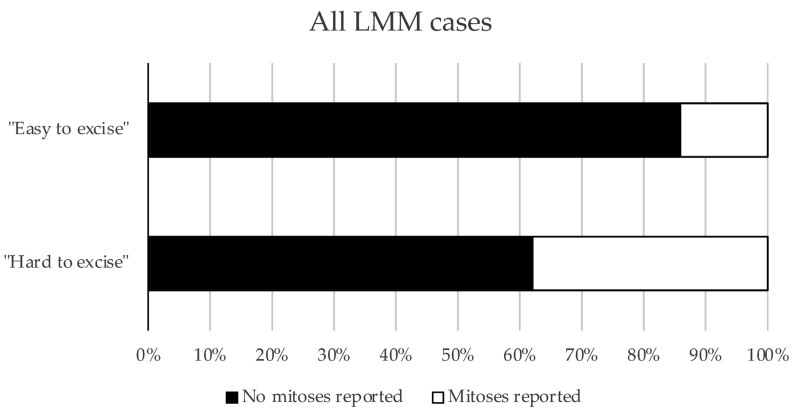
Mitoses in respective subgroups of Cohort 2. The proportions of cases with or without mitoses in the “easy to excise” and “hard to excise” LMM cases from Cohort 2 are presented as a bar graph (*p* = 0.0024, Pearson’s chi-squared test).

Notably, LMM cases that were hard to excise showed significantly more dermal mitoses than LMM cases with few surgical resections until R0 (“easy to excise”) (*p* = 0.0024) (Figure 2).

Furthermore, LMM cases with mitoses had a higher pT classification than LMM cases without mitoses (*p* < 0.001). A statistically significant increase in “hard to resect” LMM cases was confirmed in the pT2a group. Furthermore, a consistent trend towards more “hard to resect” cases was also observed in the pT1a and pT1b groups, although this did not reach statistical significance (Figure S1).

**Figure 3:**
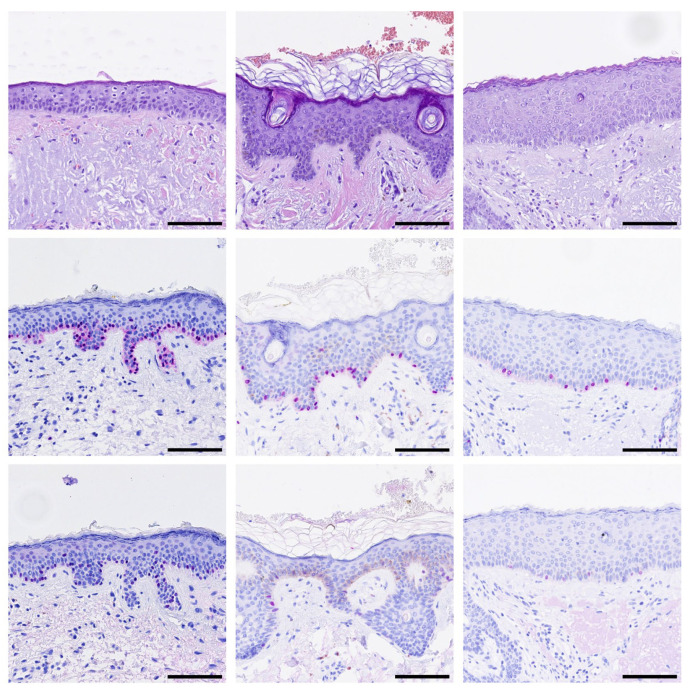
Aggregations of PRAME-positive melanocytes in safety margins. Representative images of H&E and immunohistochemistry stainings for SOX10 (red) and PRAME (red) are shown. (**Top row**): H&E, (**Middle row**): SOX10, (**Bottom row**): PRAME staining of three representative cases. Black bar = Scale bar = 100 µm.

Last, we decided to analyze 22 cases (from 22 individual patients) with LMM that have not been stained with PRAME before. All primary tumors were PRAME positive. Surprisingly, a high number of cases (36.36%, 7/22) previously labeled as “tumor-free” displayed PRAME^+^ junctional aggregates of melanocytes in safety margins that have been taken to extend tumor-free margins (Figure 3).

**Figure 4:**
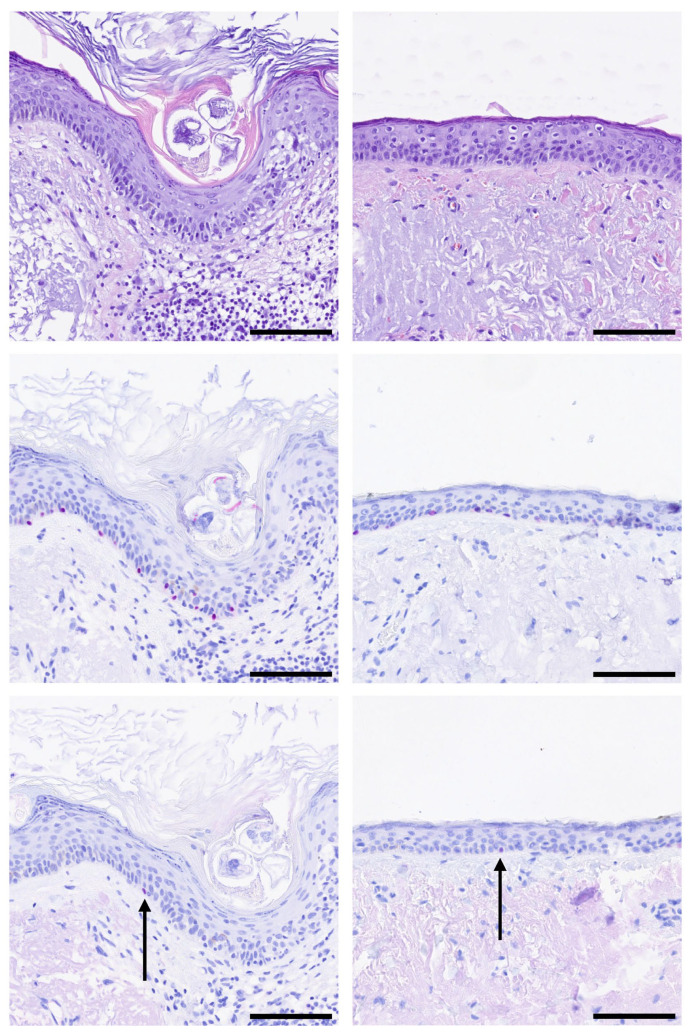
Single PRAME-positive melanocytes (black arrow) in safety margins. Representative images of H&E and immunohistochemistry stainings for SOX10 (red) and PRAME (red) are shown. (**Top row**): H&E, (**Middle row**): SOX10, (**Bottom row**): PRAME staining of two representative cases. Black bar = Scale bar = 100 µm.

There were no statistically significant differences regarding the number of PRAME^+^ aggregates between LMM cases with mitoses (3/7 in Cohort 2.1) and LMM cases without mitoses (4/15 in Cohort 2.2) (*p* = 0.4476, Pearson’s chi-squared test). Isolated PRAME^+^ single cells were found in most cases (77.27%, 17/22, Figure 4).

To assess the clinical relevance of these findings, we tried to follow up with all 22 patients from the experimental cohort in 05/2025. We were able to follow up on 18/22 patients and found no self-reported local recurrence of disease. Of special interest, 7/7 patients with aggregations of PRAME-positive melanocytes in safety margins reported no local recurrence, progressive disease, or death (median follow-up: 5 years), with the caveat of limited follow-up time and small numbers.

## 4. Discussion

In this study, we demonstrated that the reported number of mitoses correlated with more surgical procedures in a large single-center cohort from a tertiary hospital. Dermal mitoses have been implicated in worse clinical outcomes and recurrent disease in melanoma patients in general [32], but a correlation with more surgical procedures in LMM has not been reported yet. Importantly, histologic margin status was found to be the most important predictor of relapse in LMM, and a higher overall mitotic rate was found in an univariate analysis as well [33], but the cohort analyzed was diagnosed before the routine use of PRAME. Pre-operatively, the presence of dermal mitoses might be taken into account when planning surgical excisions. This data point could contribute to a more comprehensive surgical strategy that also incorporates established guidelines, patient preferences, comorbidities, tumor location, and the potential for primary wound closure.

Historically, the first paper describing the mitotic rate (MR) of primary melanomas as a relevant factor in the 1970s proposed a “prognostic index” (defined as the product of tumor thickness and the number of mitoses per square millimeter) [34]. In 1986, McGovern et al. published recommendations on melanoma reporting, which included “mitoses per mm^2^” [35]. The 2009 AJCC classification [36] included these recommendations over 30 years later, leading to widespread adaptation by pathologists. These guidelines upgraded thin melanoma ≤ 1.0 mm with ≥1 mitosis/mm^2^ to pT1b. A major point of criticism regarding MR is intra- and interobserver reproducibility, with studies reporting satisfactory [37,38] reproducibility on one hand and insufficient reliability on the other hand [39]. Interestingly, there is more robust evidence regarding the prognostic value of the MR in primary melanomas [40,41]. Nevertheless, a new consensus on the available evidence [42] led to a change in the following 2018 AJCC classification [29], such that T1a melanomas include those < 0.8 mm without ulceration, while T1b melanomas include those 0.8–1 mm with or without ulceration and those < 0.8 mm with ulceration regardless of MR. Since there is ongoing scientific debate about the utility of the MR and its impact on treatment decisions (also including assessment of MR in sentinel lymph nodes [43]), we strongly recommend reporting the MR in every malignant melanoma.

Furthermore, our study sheds additional light on the use of PRAME in resection margin assessment in LMM. PRAME-positivity in LM and LMM in our study was in line with the literature [27]. In PRAME^+^ cases from our experimental cohort, we found a large proportion of previously labeled “tumor-free” safety margins to contain nests of PRAME^+^ melanocytes in certain areas and an even higher proportion of PRAME^+^ single cells in those cases, respectively. Surprisingly, no local recurrences were observed, although in a limited follow-up. Our findings raise the question of how strict tumor margins in LMM should be judged using PRAME, taking into account that the current literature describes histologic security margins of 3 mm, equating to a clinical margin of greater than 6.5 mm [33,44]. PRAME^+^ single cells in the epidermis are a well-reported feature, especially in high CSD skin [22], while aggregates of PRAME-positive cells have not been reported as an incidental finding so far, especially adjacent to a PRAME^+^ melanoma. Whether the reported nests are part of the reported melanoma or incidental cannot be answered by this study. Molecular testing might be helpful in these cases to determine shared mutations between distant nests and primary tumors on a single-cell basis, as previously described in acral melanoma [45].

A prospective study to compare more conservative and radical surgical treatment of LM and LMM, taking margin judgement using PRAME into account, is urgently warranted. Endpoints should not only include overall and disease-free survival, but also morbidity due to surgical burden and quality of life. LMM mostly affects the elderly and is often localized at the head and neck, resulting in disfiguring surgical procedures without primary wound closure after resection with safety margins. The normal, acceptable density for PRAME-positive cells in margins has yet to be determined [46] and has to be correlated with other factors, including cell size, overall melanocyte density, and histomorphology.

With this, we hope to optimize safety margin assessment with PRAME, especially regarding the interpretation of single PRAME^+^ cells in surgical margins, and reduce morbidity after surgical treatment of LMM.

## 5. Conclusions

This study establishes a novel correlation between present dermal mitoses vs. no reported dermal mitoses and the need for more extensive surgical procedures in a large single-center cohort in cases of LMM. While the general prognostic value and reproducibility of mitotic rate in melanomas remain a subject of ongoing debate, our findings suggest another practical utility as a pre-operative indicator for surgical planning in LMM. Incorporating the assessment of dermal mitoses could contribute to a more comprehensive surgical strategy, complementing established predictors.

Furthermore, our investigation into the use of PRAME immunohistochemistry for margin assessment reveals significant challenges to current practices. We identified PRAME-positive cells within surgical margins previously considered tumor-free, yet, surprisingly, this did not correlate with local recurrence in our limited follow-up. This finding raises a critical question regarding the clinical significance of such cells and the appropriate interpretation of PRAME-positive margins. The acceptable density and morphology of PRAME-positive cells in sun-damaged skin adjacent to an LMM have yet to be determined.

Therefore, a prospective study is urgently warranted to compare conservative versus more radical surgical approaches in LMM, integrating PRAME for margin evaluation. Future research should aim to establish clear criteria for interpreting PRAME results and may benefit from molecular testing to determine the clonal relationship between primary tumors and distant PRAME-positive nests. Clinicians and pathologists should refine treatment protocols to balance oncologic safety with the reduction of surgical morbidity and improvement of quality of life, particularly for elderly patients with tumors in cosmetically sensitive locations.

## Figures and Tables

**Table 1:** Subgroup analysis of case Cohort 1 (n.s. = not significant).

Characteristic	Overall (*N* = 80)	Hard to Resect (*N* = 13)	Easy to Resect (*N* = 67)	Significance
Age at diagnosis—Mean (SD)	75.7 (11.1)	74.1 (11.4)	76.0 (11.1)	n.s.
Excisions for R0—Mean (SD)	0.6 (0.8)	2.2 (0.4)	0.3 (0.5)	(*p* < 0.001)
Sex				n.s.
Female	38 (47.5%)	8 (61.5%)	30 (44.8%)	-
Male	42 (52.5%)	5 (38.5%)	37 (55.2%)	-
Subtype				n.s.
LM	45 (56.2%)	6 (46.2%)	39 (58.2%)	-
LMM	35 (43.8%)	7 (53.8%)	28 (41.8%)	-
Ulceration				n.s.
No	79 (98.8%)	13 (100.0%)	66 (98.5%)	-
Yes	1 (1.2%)	0 (0.0%)	1 (1.5%)	-
Localization				n.s.
Arm	16 (20.0%)	5 (38.5%)	11 (16.4%)	-
Head	60 (75.0%)	8 (61.5%)	52 (77.6%)	-
Leg	2 (2.5%)	0 (0.0%)	2 (3.0%)	-
Torso	2 (2.5%)	0 (0.0%)	2 (3.0%)	-
Stage at diagnosis				n.s.
0 (LM)	45 (56.2%)	6 (46.2%)	39 (58.2%)	-
IA	23 (28.7%)	5 (38.5%)	18 (26.9%)	-
IB	7 (8.8%)	1 (7.7%)	6 (9.0%)	-
IIA	2 (2.5%)	0 (0.0%)	2 (3.0%)	-
IIB	1 (1.2%)	1 (7.7%)	0 (0.0%)	-
IIC	1 (1.2%)	0 (0.0%)	1 (1.5%)	-
IIIB	1 (1.2%)	0 (0.0%)	1 (1.5%)	-
Previous history of melanoma				n.s.
Yes	15 (18.8%)	1 (7.7%)	14 (20.9%)	-
No	65 (81.2%)	12 (92.3%)	53 (79.1%)	-
PRAME-Status				n.s.
Negative	4 (5.0%)	0 (0.0%)	4 (6.0%)	-
Positive	76 (95.0%)	13 (100.0%)	63 (94.0%)	-

**Table 2:** Subgroup analysis of case Cohort 2 (n.s. = not significant).

Characteristic	Overall (*N* = 171)	Mitoses Reported (*N* = 31)	No Mitoses (*N* = 140)	Significance
Age at Diagnosis—Mean (SD)	74.9 (10.1)	73.9 (11.9)	75.1 (9.7)	n.s.
Sex				n.s.
female	67 (39.2%)	11 (35.5%)	56 (40.0%)	-
male	104 (60.8%)	20 (64.5%)	84 (60.0%)	-
Localization				n.s.
Arm	20 (11.7%)	2 (6.5%)	18 (12.9%)	-
Head	137 (80.1%)	29 (93.5%)	108 (77.1%)	-
Leg	2 (1.2%)	0 (0.0%)	2 (1.4%)	-
Trunk	12 (7.0%)	0 (0.0%)	12 (8.6%)	-
Initial Stage				*p* < 0.001
pT1a	132 (77.2%)	11 (35.5%)	121 (86.4%)	-
pT1b	13 (7.6%)	5 (16.1%)	8 (5.7%)	-
pT2a	12 (7.0%)	2 (6.5%)	10 (7.1%)	-
pT2b	2 (1.2%)	2 (6.5%)	0 (0.0%)	-
pT3a	6 (3.5%)	5 (16.1%)	1 (0.7%)	-
pT3b	2 (1.2%)	2 (6.5%)	0 (0.0%)	-
pT4a	2 (1.2%)	2 (6.5%)	0 (0.0%)	-
pT4b	2 (1.2%)	2 (6.5%)	0 (0.0%)	-
Mitotic rate (MR)—Mean (SD)	1.0 (3.3)	5.3 (6.0)	0.0 (0.0)	*p* < 0.001
Number of procedures until margin-free resection (R0)	0.7 (1.1)	1.3 (1.6)	0.5 (0.8)	*p* = 0.0076
Hard to excise				*p* = 0.0024
no	142 (83.0%)	20 (64.5%)	122 (87.1%)	-
yes	29 (17.0%)	11 (35.5%)	18 (12.9%)	-

## Data Availability

Case data of Cohort 1 and Cohort 2 are available on reasonable request.

## Supplementary Materials

The following supporting information can be downloaded at https://www.mdpi.com/article/10.3390/cancers17193112/s1, Figure S1: Statistical analyses of subgroups in Cohort 2.

## Abbreviations

The following abbreviations are used in this manuscript:

CSD	Chronic sun damage
H&E	Hematoxylin and Eosin
LM	Lentigo maligna
LMM	Lentigo maligna melanoma
MR	Mitotic rate
pT	Pathological tumor stage
PRAME	Preferentially expressed antigen in melanoma
R0	Margin-free resections
SD	Standard deviation
SOX10	SRY-Box Transcription Factor 10
Y/N	Yes/No
