# Supplementary material for: Dermal Mitoses Correlate with Surgical Burden in Lentigo Maligna Melanoma: PRAME for Margin Assessment
Source: Cancers (Basel). 2025 Sep 24;17(19):3112. doi: 10.3390/cancers17193112 (PMC12523903; doi:10.3390/cancers17193112)
Supplement: Supplementary file 1 [file cancers-17-03112-s001.zip › cancers-3882834-supplementary.pdf]

Supplementary Materials

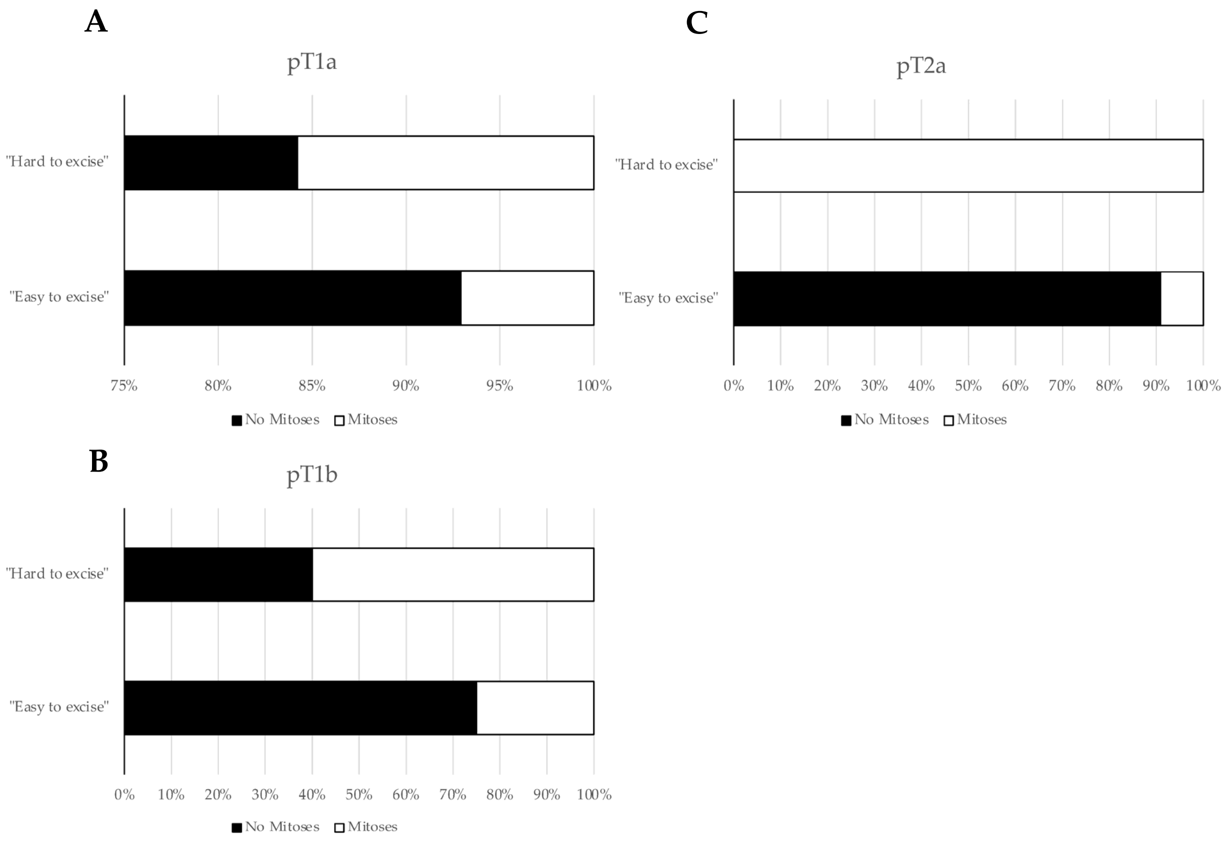

Figure S1. Subgroups according the top T stage.

Proportions of „Easy to Excise“ (lower bar) and „Hard to Excise“ (upper bar) cases in relation to Mitoses reported (No mitoses reported: black bars, Mitoses reported: white bar) according to the top T status (**A**: pT1a, p=0.2038, n=132; **B**: pT1b, p=0.207, n=13; **C**: pT2a, p=0.0195, n=12)

pT2b, pT3a, pT3b, pT4a and pT4b were omitted due to small numbers (N<7 in each category)
